# The Function and Molecular Mechanism of Commensal Microbiome in Promoting Malignant Progression of Lung Cancer

**DOI:** 10.3390/cancers14215394

**Published:** 2022-11-02

**Authors:** Haiyang Wang, Jiayi Hu, Junlu Wu, Ping Ji, Anquan Shang, Dong Li

**Affiliations:** Department of Laboratory Medicine, Tongji Hospital of Tongji University School of Medicine, 389 Xincun Road, Shanghai 200065, China

**Keywords:** lung cancer, dysbiosis, microbiome, carcinogenesis, immune response

## Abstract

**Simple Summary:**

The commensal microbiome in the human body must always be considered as a potential factor in carcinogenesis that may play a crucial role in the occurrence and progression of lung cancer. Herein, we give great details about the biological processes involved in lung cancer mediated by the microbiome. Potential mechanisms include regulating host immune activity by means of a variety of pathogenic factors, dysregulating host metabolism as a result of microbiome alterations, and microbiome dysbiosis, which are well elucidated in this article.

**Abstract:**

The human commensal microbiome existing in an internal environment is relatively consistent with that of the host. The presence of bacterial dysbiosis, on the other hand, promptly results in the termination of this symbiotic association. The altered microbial structure in the lung may be responsible for the development of lung cancer by controlling the host’s inflammatory response and influencing a variety of immunological pathways. More and more studies have pointed to the fact that the commensal microbiota plays a vital role in both the development of tumors and the body’s response to lung cancer treatment. Microbiome dysbiosis, genotoxicity, virulence effect, and epigenetic dysregulations are some of the potential mechanisms that may lie behind the process of tumorigenesis that is mediated by microbiome. Other potential mechanisms include regulating host immune activity through a variety of pathogenic factors, dysregulating host metabolism as a result of microbiome alterations, and microbiome dysbiosis. In this historical overview, we go through some of the more recent mechanistic discoveries into the biological processes that are involved in lung cancer that are caused by bacteria. Without a question, obtaining a greater knowledge of the dynamic link between the lung microbiome and lung cancer has the potential to inspire the development of innovative early detection and customized treatment methods for lung cancer.

## 1. Introduction

Epithelial surfaces exposed to the outside world are constantly inhabited by many microorganisms, including bacteria, archaea, viruses, and fungi, all of whom are part of the “commensal microbiome” [1,2]. These microbiome control how nutrients are utilized and prevent the epithelial lining of the gut from being damaged. Over time, the commensal microbiome and the host enter into a mutually beneficial relationship that leads to the formation of a dynamic micro-ecosystem [3]. When these microorganisms become unbalanced, it may lead to various diseases. This suggests that the structural integrity of the intestinal mucosa and the balance of intestinal flora are associated with a variety of disorders [4,5]. Despite the fact that the human microbiome has coevolved with the host and is required for many biological processes of the human body [6], the type and amount of human bacteria in the gut are closely related to various types of tumors.

Lung adenocarcinoma (LUAD), the most prevalent kind of cancer disease, is the primary cause of cancer-related mortality globally. Therefore, there is an urgent and societal need to learn more about the causes of cancer and its treatment. Although much is known about the genomic landscape of LUAD, less is known about the factors outside of tumor cells that control lung cancer growth. Lung cancer has long been believed to be a complex illness brought on by interactions between the host and its environment [7]. Commensal bacteria directly impact how tumors form, grow, and respond to treatment [8,9]. As the biggest mucosal tissue with the highest surface area in the body, the lungs are exposed to a wide range of airborne microorganisms and environmental stressors during breathing. Therefore, the connection between lung cancer and the host’s reaction to microbiome is particularly intriguing.

The gut bacteria and colorectal cancer (CRC) have been extensively studied because the human gut is a natural reservoir of bacteria. However, the relationship between bacteria and lung cancer has not been studied as much as the relationship between gut bacteria and CRC. This is due to the fact that a healthy lung is sterile. However, a number of new studies demonstrating the heterogeneity of lung microbiome and its link to pulmonary diseases and lung cancer have cast doubt on this paradigm [10].

Numerous studies have focused on the distinctions between microbiome in tumor tissues and the function of intratumor or tumor-associated bacteria [11,12]. Immune checkpoint inhibitors are less effective when antibiotics are administered, but outcomes are improved when certain strains of gut bacteria are present or there are a greater number of them [13,14]. Additionally, the bacterial populations that reside exclusively within tumors are tumor-type specific, indicating a link with tumor growth [15]. Considering all previous research, we can conclude that the naturally occurring bacteria in our bodies have two distinct effects on lung cancer, including the inhibition and promotion of malignant progression. Extensive research has been conducted on the systemic effect of gut bacteria on cancers of the GI tract and elsewhere [16,17]. In this study, we discuss the composition and differences of microbiomes in the respiratory tract, lung, and gastrointestinal tract, with a focus on the activities and mechanisms of bacteria in response to lung cancer.

## 2. Microbiomes in the Lung, Gut and Respiratory Tract

**Figure 1:**
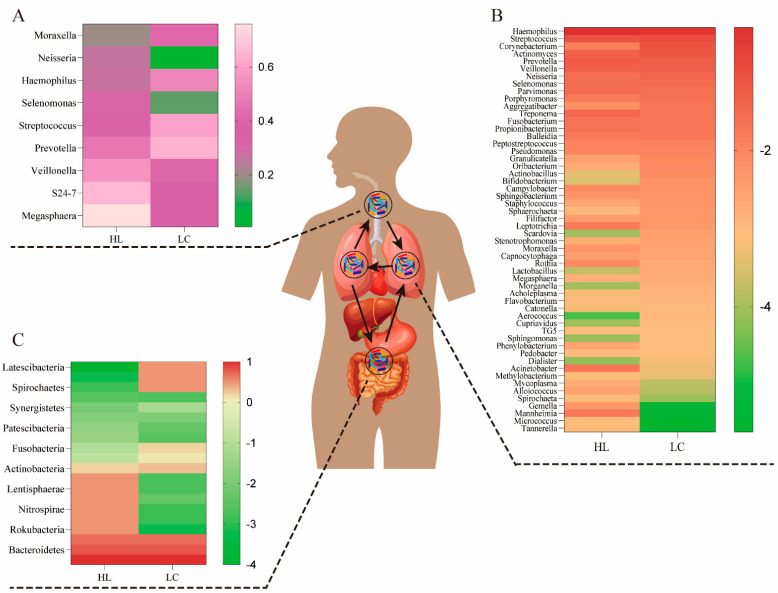
The bacteria composition of the airway, lung, and gut in healthy (HL) and lung cancer (LC) individuals. (**A**) The airway bacteria composition of the top ten taxa at the genus level. (**B**) The composition of lung bacteria at the genus level. (**C**) The gut bacteria composition of the main taxa at the phylum level. The oral bacteria may be the primary source of lung bacteria due to a long-term and dynamic circulation (through mucosal dispersion, swallowing, and micro-aerosols or secretions generated in oral cavities).

The lungs are connected to the external environment through the trachea and oral cavity. Thus, when lung cancer occurs, there may be a connection between the bacterial species and the balance of bacteria in the lungs, airways, and gut. The microbiome of the mouth, lung, and intestine can communicate directly through the mucosal spread, respiration, and digestion, and indirectly through the circulation of inflammatory substances, cytokines, and metabolites (Figure 1). *Prevotella*, *Streptococcus*, *Megasphaera*, and *Veillonella* are the most abundant microbiome in the airways, and compPLS analysis revealed that *Veillonella* and *Megasphaera* are associated with upregulation of cancer signaling pathways [18]. Researchers have found that oral commensals likely enter the lower respiratory tract through a process called “micro-aspiration” [19]. Thus, the bacteria in the lower respiratory tract are a reflection of both the bacteria in the lungs and the bacteria in the mouth. *Enterococcus*, *Veillonella*, *Agathobacter*, *Megasphaera*, and *Coriobacteriaceae* were the most common microbiomes in the viscera [20], and their abundance in lung cancer groups was significantly higher than their counterparts in healthy groups. The most prevalent taxa in the lungs have been identified, including *Firmicutes*, *Bacteroides*, and *Proteobacteria*, as well as genera such as *Streptococcus*, *Veillonella*, *Pseudomonas*, and *Prevotella* [21]. Below, we conduct a systematic summary and comparison of the above organs and their bacterial diversity.

### 2.1. Lower Airway Microbiota

In a small cohort research, *Veillonella* and *Megasphaera* were shown to be abundant in the lower airways of lung cancer patients [22]. *Capnocytophaga*, *Selenomonas*, *Veillonella*, and *Neisseria* were significantly altered in lower airway samples from patients with squamous cell carcinoma and adenocarcinoma compared to healthy controls [23]. Recent research suggests that unique lung bacteria detected in the lower respiratory tract influence the host immunological profile. The presence of oral anaerobic taxa, including *Veillonella* species, in the lower airway microbiome is related with increased infiltration of inflammatory cells T helper type (Th17) cells and activation of the ERK/PI3K pathway in bronchial epithelial cells, as described by Tsay et al. [18]. In particular, ERK/PI3K activation in bronchial epithelium is an early event in the development of lung tumors, and its dysregulation is associated with disease progression [24]. Recent evidence suggests that dysbiosis of lower airway bacteria may influence lung carcinogenesis via multiple mechanisms, including the upregulation of inflammatory pathways in the host, the production of bacterial toxins that alter host genomic stability, and the release of carcinogenic microbial metabolites [5,25]. A random forest classifier used for lower respiratory tract data revealed that *Prevotella*, *Veillonella*, and *Streptococcus* were the most common taxa predictive for lung cancer. In addition, *Megasphaera* and *Veillonella* [26,27], which are typically found in the phenotype, were the most common taxa found to be strongly associated with cancer-related pathways. Notably, A549 cells treated in vitro to the aforementioned bacteria (including *Veillonella*) or their bacterial metabolites displayed elevation of upstream pathways in the ERK, IL-17, PI3K, and VEGF signaling pathways and downregulation of the PTEN (phosphatase and tensin homolog) pathway. Thus, previous research has demonstrated that there is a strong correlation between the presence of *Veillonella* in the lower respiratory tract and lung cancer, and that the transcriptomic alterations were predominantly driven by microbial products [18]. We list the proportions of representative genera of the airway microbiome in the presence or absence of lung cancer in Figure 1A.

### 2.2. Lung Microbiota

The microbiome of the lung consists of bacteria, fungi, and viruses generated from mucosal secretions, the nasopharynx, the oropharynx, and air exchange [26,28,29]. On the basis of long-term and verified data (through mucosal dispersion, swallowing, and micro-aerosols or oral secretions), some researchers have argued that the oral bacteria may be the major source of lung bacteria [30]. The respiratory tract and gastrointestinal tract could communicate through physiological systems such as micro-aspiration and inhalation [31]. Under healthy conditions, these microbiomes and lung tissues maintain a healthy balance, and they do not induce respiratory infections. However, this is not unexpected when multiple unidentified interactions in other tissues are taken into account. All of the above interplay among the gut bacteria, immunity, and metabolism in these microbial niches affects the pathogenesis of multiple lung diseases, such as idiopathic pulmonary fibrosis, cystic fibrosis (CF), and lung cancer (Table 1) [27,32]. CF is a progressive hereditary illness caused by genetic abnormalities in the CF transmembrane conductance regulator (CFTR) protein. Several studies have shown that the varied microbiota in CF may lead to CF phenotypic differences [33]. The presence of *Moraxella catarrhalis*, *Haemophilus influenzae*, or *Streptococcus pneumoniae* in the hypopharynx has been related with an increased risk of pediatric asthma [34]. Alpha diversity is much greater in nonmalignant lung tissues than in lung tumor tissues, according to accumulating data [35]. In order to acquire a more intuitive grasp of the relationship between lung bacteria composition and the development of lung cancer, Figure 1B illustrates the lung bacterial composition of a healthy lung and a lung with lung cancer.

### 2.3. Gut Microbiota

The microorganisms that populate the digestive system are referred to as gut bacteria. Gene expression in these organisms is 150 times greater than in human cells, despite the fact that the overall number of gut bacteria cells is equivalent to that of human cells. Despite the advances achieved in examining the association between the gut microbiome and lung cancer patients, the structure and functional importance of these taxa remain largely unclear [36]. Individual differences in the characteristics of the gut bacteria in lung cancer patients suggest that the gut bacteria may have a role in the therapeutic and prognostic aspects of lung cancer [13]. There is accumulating evidence that the development of lung cancer is driven by an interplay of hereditary and environmental variables [41]. Recent research has shown that lung cancer incidence and development are also related to human gut bacteria. This is because the interaction among these organisms affects the functioning of various metabolic, inflammatory, and immune pathways [42,43]. Neoplastic diseases of the lung are characterized by a number of common features, one of which is an alteration in the amount of intestinal flora [44]. Some intestinal flora species including *Fusobacterium nucleatum*, *Escherichia coli*, *Bacteroides fragilis*, and *Aspergillus* have been linked to the development of cancer [44]. The prevalence of Veillonella, Bacteroides, and Clostridium was significantly higher in lung cancer patients than in healthy individuals, according to a previous study [45]. One prior research indicated that the number of butyrate-producing bacteria in the intestines of non-small-cell lung cancer (NSCLC) patients decreased dramatically [46]. According to the results of Zhao et al., Erysipelotrichaceae and Phascolarctobacterium in gut were substantially more abundant in the healthy group than in the lung cancer group [20]. Erysipelotrichaceae is one of the major butyrate-producing bacteria in the intestines [19], while the Phascolarctobacterium is involved in the production of short-chain fatty acids (SCFAs) [47]. This alteration was precisely proportionate to the feedback impact of the “lung–gut axis” on the immune response in the distal lung [48]. Therefore, we summarized the representative microbiome proportions in the gastrointestinal tracts of an individual at the genus level based on the presence and absence of lung cancer (Figure 1C).

## 3. Main Mechanisms of Microbiome Carcinogenesis

**Figure 2:**
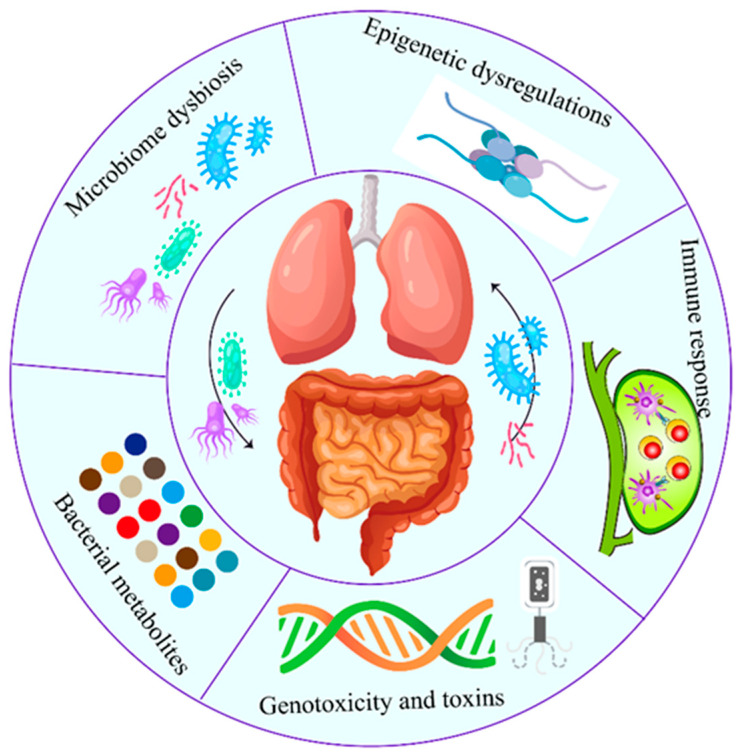
Role of airway–lung–gut axis in the lung cancer pathogenesis. Both local (lung) and distal (gut and airway) microbiota play critical roles in lung cancer. The carcinogenic process induced by microbiome involves five established mechanisms. Specifically, all five mechanisms interact and influence each other during the carcinogenesis.

**Figure 3:**
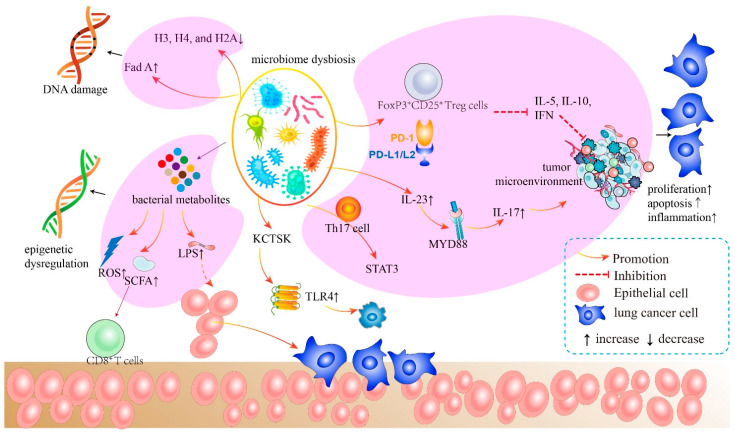
The mechanism of bacteria of the airway–lung–gut axis in lung cancer pathogenesis. The microbiota generates cytotoxicity-related components, which induce host cell DNA damage, epigenetic dysregulation, and aberrant immunological pathways. The microbiota and its metabolites activate LPS, leading to subsequent inflammatory responses. These inflammatory activators initiate crucial downstream signaling pathways that promote malignant activity in host cells.

The correlation between intratumor microbiome carcinogenesis and the progression of lung cancer has been established. In general, five primary mechanisms have been demonstrated as potential mechanisms of action, which are discussed individually below (Figure 2). Specifically, all these five mechanisms interact and influence each other during carcinogenesis (Figure 3).

### 3.1. Immune Responses Modulated by Microbiome

The microbiome plays a major role in shaping adaptive immunity throughout an individual’s lifetime. By modulating host susceptibility to numerous pathogenic factors and therapeutic outcomes, the microbiome of the lung and gut affects the immunological activity of the host either directly or indirectly. In contrast, the condition of the microbiome is altered and perpetuated by the host immune system and external variables. A balance between the populations of common bacterial species is maintained, and the development of harmful bacteria is inhibited. Consequently, it is plausible that changes in the microbiome might regulate the immune response of the host. Therefore, a comprehensive understanding of the immune response and inflammatory pathways controlled by the bacteria is required. The connection among the microbiome, inflammation, and the immune system allows the host to detect and inhibit bacterial or fungal invasion and infection. The significance of the microbiome’s control of immune responses in cancer must, thus, be stressed.

A recent study investigated the impact of the gut microbiome on immune protection in lung cancer. *Enterotoxigenic Bacteroides fragilis* (ETBF) activates STAT3 through a selective Th17 response in mice, suggesting that human commensal bacteria may promote cancer through a Th17-dependent mechanism [49]. Li et al. discovered that, in microenvironmental disruption induced by intestinal dysbacteriosis, tumor-secreted metastasis-related secretory protein cathepsin (KCTSK) can bind to Toll-like receptor 4 (TLR4), to stimulate M2 polarization of tumor-associated macrophages (TAMs) via an mTOR-dependent pathway, which, in turn, promote the invasion and metastasis of NSCLC cells through the NF-κB pathway [50]. This work demonstrates that both the bacteria colonizing in the lung and the flora-stability in the guts play important roles in regulating the normal antitumor immune response. Specifically, pathogenic bacteria, such as influenza, increases epithelial IL-17C production in chronic obstructive pulmonary disease (COPD) patients, thus enhancing tumor growth through neutrophilic inflammation in the tumor microenvironment [51]. The lung bacterium influences the production of innate immunity genes such as IL-5, IL-10, and IFN, and the expression of PD-L1 on CD11bC DCs and FoxP3^+^CD25^+^ Treg cells was elevated in the lungs of SPF (specific pathogen-free) neonates compared to GF (germ-free) mice [52]. Furthermore, commensal bacteria may modify immunity in the airway mucosa through inflammasomes and give immunological activation signals following influenza virus infection [53]. The enrichment of the lung microbiome with oral taxa has been shown to be related to Th17 inflammation, with the makeup of the lung microbiome impacting TLR4 responses. In addition, researchers have shown in a preclinical model that modification of the gut microbiome might modify the immunological responses of the host and susceptibility to lung infection [53,54]. In preclinical studies, germ-free (GF) mice without gut flora exhibited severe immunological abnormalities with an inadequate mucous layer, an immunoglobulin production abnormality, and a reduction in lymph node size and quantity [55]. This is in part because the reduced IgA generated by bacteria is unable to regulate bacterial pathogenicity in the gut by inhibiting bacterial adhesion to mucosal epithelial cells, thus dampening the body’s innate immune response [56]. Dzutsev et al. hypothesized that lung cancer patients with bacteria deficits may react poorly to immunotherapy owing in part to alterations in the bacterial structure that help define the host’s immune system [57]. 

In addition, high levels of commensal bacteria may improve the clinical efficacy of vaccines [58], underscoring the importance of the microbiome. In this multiple interaction, activation of inflammatory pathways, such as microbe-associated molecular patterns (MAMPs) or pattern recognition receptors (PRRs), not only senses the status of the bacteria, but also triggers proliferation and survival of epithelial cells under certain circumstances, thereby promoting cancer development [59]. Other results suggested that TLRs activated by bacteria play a curative role in cancer development in colon, stomach, liver, and pancreas. Moreover, MYD88 induced by bacteria triggers IL-23 signaling in myeloid cells to enhance tumor progression and the formation of tumoral IL-17 responses [60]. It is well known that deficiency of IL-17C promotes proliferation and metastasis of cancer cells [51]. A growing body of research suggests that nucleotide-binding oligomerization domain-like receptors (NOD) are a membrane-localized subfamily of PRRs. NOD1 provides protective benefits by acting as a barrier to prevent the transition from inflammation to carcinogenesis [61], while NOD2 modulates the bacteria and reduces susceptibility to CRC [62]. Similar results were observed in NOD-deficient mice, which plays a critical role in microbiome dysbiosis and carcinogenesis [63]. Taken together, the aforementioned studies suggested many cascaded immune pathways directly or indirectly modulated by bacteria.

### 3.2. Inflammataory Pathways Modulated by Bacteria

Intestinal bacteria not only impact inflammatory responses at the local mucosal level, but also cause chronic pulmonary inflammation through gut–lung axis communication, according to a growing body of research. Likewise, the inflammatory response caused by lung microorganisms often occurs directly on mucosal surfaces. Pathogenic bacteria produce inflammation and increased mucus production during first infection. This results in injury to the respiratory epithelium, which subsequently inhibits the innate immune response and enhances the inflammatory response. The weakened immune response renders the lung susceptible to infection by harmful germs, thus initiating a vicious cycle. Huang et al. found that Proteobacter taxa including *Enterobacteraceae*, *Pasteurellaceae*, and *Bacillaceae* are associated with Th17-related gene signature [64]. In individuals with pulmonary illness, the Th17 inflammatory phenotype may represent another mechanism independent of the Th2 response. Notably, the Th17 inflammatory pathway and IL-17 overexpression may not be the only ones elevated in a lung dysbiosis host. A recent study indicated that, in pIgR-deficient animals, lack of mucosal immunity leads to pathogen penetration into the epithelium, causing a severe inflammatory response and further increasing bacteria-induced lung infection. Moreover, under situations such as chronic inflammation, *Gammaproteobacteria* may outcompete bacteria that cannot survive by metabolizing inflammatory metabolites. Under inflammatory circumstances, *Gammaproteobacteria* may utilize reactive nitrogen species, a result of many inflammatory cells, as a terminal acceptor for electrons to sustain development. Consequently, it is conceivable that the altered microenvironment induced by potentially harmful bacteria would promote continuous or chronic inflammation, thereby accelerating the progression of lung cancer [65,66,67].

Although many researchers think that the inflammatory pathways induced by bacteria maybe comparable with immune pathways, the detailed mechanism underlying the carcinogenesis is obscure compared with immune response. The involvement of regulatory T-cell subsets and TLRs, inflammation cytokines and mediators, surfactant protein D, and several other factors have been proposed as the underlying mechanisms [68]. However, owing to the presence of easily detectable inflammatory factors in peripheral blood, such as C-reactive protein (CRP), tumor necrosis factor-alpha (TNF-α), and IL-6, lung illnesses caused by bacteria may be recognized sooner. In particular, CRP is positively correlated with the abundance of gut bacteria in lung cancer patients, indicating that the elevated level of inflammatory components in peripheral blood may serve as a risk indication for lung cancer-mediated bacterial dysbiosis [69].

### 3.3. Host Metabolism and the Bacterial Metabolites

Bacterial metabolites are more complicated in content and structure than host metabolites, which can affect the differentiation propensity of naïve T cells, effector T cells, Tregs, or the release of Th17, further triggering systemic inflammation and immune response. Intestinal dysregulation of host metabolism by alterations of the microbiome has been actively explored [70,71]. The resulting metabolites play an important role in regulating various physiological activities in humans and can influence genotoxic or tumor suppressive capabilities through various methods, such as providing metabolic energy, stimulating biosynthesis, and altering signaling proteins [72]. Therefore, disruption of the metabolic balance caused by disturbed homeostasis of the microbiome may promote tumor formation. Furthermore, it has been postulated that this process is triggered by the formation of carcinogenic metabolites by inhibiting immune responses and altering the host inflammatory response. Therefore, the study of the metabolome of the gut microbiome is potentially useful for cancer detection. This is primarily due to the advent of high-throughput methods, which have revolutionized molecular and genetic studies and resulted in the discovery of a number of cancer biomarkers and other very complex interspecies interactions.

The spectrum of metabolites produced by gut bacteria permeates the circulatory system and regulates the physiopathological status of distant organs, such as the lungs. The gut–lung axis, in which the lungs respond to enteric flora-derived components in peripheral blood, is one example [73]. The microbiome-produced carcinogens acetaldehyde and deoxycholic acid have been linked to esophageal and liver carcinogenesis [74]. Lipopolysaccharides (LPS), one of the endotoxins produced by the outer membrane of Gram-negative bacteria in the intestine, could affect the lung response of asthma patients to allergens [75]. According to prior study, certain metabolic profiles coupled with bacterial species were shown to be associated with the glycerophospholipid and lineolate pathways, which play a key role in the development of pneumonia in HIV-infected persons [76]. Bei et al. observed that the combination of *Pseudomonas aeruginosa*’s primary metabolites and *Rothia mucilaginosa*’s substrates may contribute to the etiology and progression of CF [77]. SCFAs, one of the most important metabolites, are produced in enormous amounts by commensal microorganisms and play a crucial role in molecular signaling pathways. Numerous studies have investigated the role of SCFA in the gut and host immune system, but the role of SCFAs in the respiratory system, epithelium, and immune system is still unknown. A recent study showed that the fermentable dietary fiber inulin can alter the structure of the gut bacteria and its associated metabolites, such as SCFA, which ultimately improves the response of mice to influenza virus infection by reducing neutrophil-induced damage and enhancing CD8^+^ T-cell antiviral responses [78]. In addition, recent research suggests that dietary fiber facilitates the fermentation of SCFA by the gut bacteria [79]. SCFAs have anti-inflammatory properties and reduce the risk of colon and breast cancer. Several studies have shown that the bacteria lead to the production of acetaldehyde [80], a major carcinogen. On the other hand, an obesity-induced metabolite of the gut microbiome, deoxycholic acid (DCA), leads to the development of hepatocellular carcinoma associated with obesity [81]. DCA may also promote histone deacetylase-like 3 (HDAC3) through inositol triphosphate, which contributes to intestinal homeostasis and repair.

### 3.4. Microbiome Dysbiosis

Microbiome dysregulation, which is characterized by altered bacterial composition, abundance, and diversity, has been linked to an increased host susceptibility to pathogenic infections, a worsening of intestinal inflammation and autoimmunity, the onset of metabolic disorders, and the promotion of the development of neurological diseases [19]. In contrast, dysbiosis of the microbiome leads to a decrease in commensal microorganisms and an increase in inflammation-causing bacteria, which can drive carcinogenesis at multiple levels. Notably, carcinogenesis of lung cancer is thought to be the result of dysbiosis rather than specific pathogen activity [13]. Dysbiosis has been linked to an increase in genotoxins and metabolites related to mutagenesis and carcinogenesis, as well as a dysregulation of the immune system [82]. For example, in an experiment in which mice with different gut bacteria compositions were irradiated, dysbiosis had a greater impact on host cell tolerance to DNA-damaging agents [83]. Gui et al. [46] observed dysbiosis of butyrate-producing gut bacteria in people with non-small-cell lung cancer. Moreover, butyrate directly enhances the antitumor cytotoxic effect of mouse CD8^+^ T lymphocytes in vivo and in vitro by boosting the IL-12 signaling pathway in an ID2-dependent manner. Hence, it is important to maintain the balance of the bacteria colonized in the lung to promote the stability of the tumor microenvironment and normal lung tissue.

Multilevel barriers and the immune system support the symbiotic relationship between the host and microbiome [84,85]. As soon as the barrier defects or immunodeficiencies disappear, microbiome composition and bacterial translocation are disrupted, resulting in pathological interactions between the microbiome and epithelial cells or the immune system [86]; once the barrier defects or immunodeficiencies reappear, the symbiotic relationship between the host and the microbiome may exacerbate dysbiosis and subsequent chain reactions leading to carcinogenesis. Previous research has demonstrated that altering the gut bacteria of mice can alter the immunotherapeutic and chemotherapeutic response of malignant tumors. The gut–lung axis plays a crucial role in influencing lung immune responses via the inhibitory effects of histone deacetylases (HDACs) of SCFAs, providing support for the hypothesis that dysbiosis at the gut–lung axis may impair antitumor immune responses and alter them in favor of lung carcinogenesis [48,87].

Inflammatory signal activation, dietary modifications, infections, and NOD2 deficiency may also contribute to dysbiosis [61]. Dysbiosis of the microbiome results in a decrease in commensal bacteria and an increase in bacteria that cause inflammation, which can stimulate carcinogenesis on multiple levels. Recent studies have shown that alterations in the lung microbiome and dysbiosis of the respiratory bacteria play a crucial role in the development and progression of lung cancer [42]. After analyzing the taxonomic composition of bronchoscopic samples from lung cancer patients, researchers discovered an increased presence of some Gram-negative bacteria, such as *Haemophilus influenzae*, *Enterobacter* spp., and *E. coli* [88], indicating a chain reaction of dysbiosis among different parts. Sobhani et al. demonstrated that gene methylation provides the link between dysbiosis and cancer [89]. Correlations between dysbiosis and histological and DNA results in the animals were found; however, the probable involvement of dysbiosis and inflammation at the start of cancer in the colonic mucosa was not clarified [90]. Similar to melanoma patients, there appears to be a variety of effects of gut bacteria dysbiosis in response to immune checkpoint inhibitor therapy in lung cancer patients. In addition to lung cancer, dysbiosis in the stomach may directly enhance oncogenic signaling in the pancreas [91]. In pancreatic ductal adenocarcinoma (PDAC), Pushalkar et al. demonstrated migration of bacteria from the gut to the pancreas and a time-dependent relationship between gut dysbiosis and *Kras* activation [91,92].

### 3.5. Genotoxicity and Virulence Effect

There are at least 100 times more unique genes in the commensal microbiome than in the human host genome. The human organism should be considered as a “meta-organism” with complex interaction dynamics among the environment, nuclear DNA, mitochondrial DNA, and the microbiome genome [93]. The imbalance and/or changes in the composition of the bacteriome may lead to the formation of a series of toxins that stimulate the generation of free radicals by histocytes or bacteria. These radicals have a carcinogenic effect on the host organism. Disturbances in bacterial balance caused by factors such as aging or exposure to xenobiotics could trigger the development of antimicrobials or a cocktail of toxins that target rivals [94]. Consequently, microbiome-induced and/or mediated genotoxicity and virulence is also the primary mechanism via which a carcinogenic role is developed.

Only three genotoxins among a large number of bacterial toxins are known to directly affect the DNA integrity of host biological target cells [40]. Among these are typhoid toxin (TT) produced by Salmonella enterica serovar Typhi, a cytotoxic distension toxin (CDT) produced by a number of Gram-negative bacteria (*Helicobacter sp.*, *Escherichia coli*, *Shigella dysenteriae*, *Haemophilus ducreyi*, and *Campylobacter jejuni*) [95], and colibactin *Escherichia coli* strains belonging to the phylogenetic group B2 [96]. After the toxin introduces genotoxicity into the nucleus of the target cell, it generates DNA breaks and triggers the classical damage response (DNA damage response, DDR), which stops the cell cycle or causes cell death depending on the type of genome damage or toxin concentration [97]. These studies demonstrated that both harmful and “neutral” (symbiotic, commensal) bacteria are capable of causing or controlling mutations in the host organism’s cells.

Recent evidence shows that genes of the gut bacteria are capable of producing estrogen-metabolizing enzymes called the estrobolome. This presents a new challenge for studying the effects of the bacteria on the host estrogen/testosterone balance, which is essential for a number of cancer mechanisms [98]. Previous studies have implicated reactive oxygen species (ROS) as mediators of DNA damage responses. Notably, bacterial dysbiosis would alter ROS levels, resulting in DNA damage and carcinogenesis. Bacterial toxins, such as cytolethal distending toxin (CDT) [99], cytotoxic necrotizing factor 1 [100], and *Bacteroides fragilis* toxin, have been identified as agents that induce double-stranded DNA damage responses [101]. In addition, it has been demonstrated that hydrogen sulfide and superoxide radicals produced by bacteria are responsible for chromosomal instability [102]. In addition, *Fusobacterium nucleatum*-produced Fad A regulates catenin signaling by interacting with E-cadherin [103]. Burns et al. discovered an amplification of virulence-associated bacterial genes in the microenvironment of colorectal cancer, which may depend on the genomes of *Fusobacterium* and *Providencia* [104]. In addition, some lung bacteria can transfer plasmids with genetic traits to tumor cells via exosomes and exert carcinogenic functions such as drug resistance and proinflammation. Deoxycholic acid and lithocholic acid are secondary metabolites formed by intestinal bacteria from bile acids that cause DNA damage and cancer onset. The formation of harmful metabolites in the lungs due to metabolic imbalance may contribute to the development of lung cancer.

Accumulating evidence has revealed that radiotherapy in lung cancer patients not only destroys normal tissue cells and the lung bacteria, but also causes radiation-induced toxicity. This increases the susceptibility of lung cancer patients to infections with *Escherichia coli*, *Staphylococcus aureus*, *Pseudomonas aeruginosa*, and *Staphylococcus epidermidis*. Encouragingly, Chen et al. [105] discovered that fecal bacteria transplantation (FMT) could reduce radiation-induced damage, decrease oxidative stress, and improve lung function in mouse models. Through activation of MAPK/NF-κB signaling and secretion of prostaglandin F2α (PGF2α), high-level gut bacteria can suppress apoptosis of healthy lung cells.

### 3.6. Epigenetic Dysregulations Induced by Bacteria

Lung cancer is characterized by epigenetic dysregulation that is associated with genetic variations and plays a crucial role in cancer genesis and progression [106]. Epigenetic regulation is connected to the microenvironment, and, as the lung is continually exposed to environmental harm and a bacterial environment, it is more susceptible to epigenetic alterations that promote carcinogenesis. DNA hypermethylation may be an early event in the progression of lung cancer, since it is associated with the regulation of many tumor suppressor genes in lung cancer and is controlled by the microbiome [107]. Increasing evidence suggests a connection between epigenetic dysregulation and microbiome dysbiosis in lung cancer [108].

With the proliferation of opportunistic pathogens in malignancies, especially lung cancer, many enzymes and factors that maintain chromatin structure are altered. It has been observed that bacteria and their metabolites can influence the epigenetic modification of host cells to enhance their survival, replication, and resistance to the host innate immune system [109]. Activation of TLR4 by bacterial LPS leads to the entry of NF-κB into the nucleus and the production of a number of genes involved in inflammation. Under conditions of epigenetic and transcriptional equilibrium, early response genes are rapidly transcribed. However, further signal transduction and chromatin remodeling are required to activate late response genes. Symbionts and pathogens can regulate host inflammation and other cell functions by reshaping the chromatin landscape, so as to colonize and replicate in the host [110]. Bacterial metabolites (such as lipopolysaccharide) retained in the human body induce chromatin remodeling at inflammatory gene loci via a series of biochemical reactions to check the inflammatory response [111]. Recent research indicates bacterial-induced modifications of noncoding RNAs and their function in altering chromatin topology [112]. Recent research by Hu et al. [113] investigated the possible link between oncogenic viruses and lung tumors, including Jaagsiekte sheep retrovirus (JSRV), Merkel cell polyomavirus (MCPyV), Epstein–Barr virus (EBV), human papillomavirus (HPV), and John Cunningham virus (JCV). Thus, in addition to bacteria, other human microorganisms such as viruses and fungi, may also mediate the epigenetic modification of tumor-related genes and reshape the chromosome structure to ensure their better proliferation [114].

In contrast to the gut microbiome, the molecular processes driving the epigenetic changes mediated by the lung microbiome are not well understood. However, bacterial metabolites such as butyrate, biotin, and folate are thought to play an important role in this process due to their epigenetic modifying abilities [115]. Folate can be secreted by a variety of bacteria, including *Lactobacillus* and *Bifidobacteria*, and it contributes to the production of 6-methyltetrahydrofolate, a methyl group donor that influences DNA methylation [116]. Biotin-producing bacteria that colonize the lungs are necessary for the maintenance of biotinylated proteins, including biotinylated histones. Biotinylation of histones (H3, H4, and H2A) regulates DNA damage responses, gene silencing, and cell proliferation [117]. The acetylation and deacetylation of histones are critical processes for controlling transcription by enabling chromatin accessible to various transcriptional factors. The microbiome-derived acetyl group is required for the production of acetyl-CoA, a histone acetylation donor [115]. Taken together, understanding the molecular processes of bacteria-triggered epigenetic dysregulations that contribute to lung carcinogenesis requires extensive research.

## 4. Conclusions and Future Perspectives

Recent breakthroughs in the development of molecular tools to analyze bacterial genomes and the use of next-generation sequencing techniques have led to revolutionary findings in the domains of general and medical microbiology and metagenomics. This is helping to advance studies of lung bacteria, lung cancer, and their possible intrinsic link. Increasing data suggest that the lung contains a complex and varied spectrum of microorganisms that react to host and environmental factors. However, compared to the gastrointestinal system, the lung microbiome contains fewer microorganisms but demonstrates a large variety [118]. 

The bacterial metabolites of the digestive tract are complicated and diverse. Moreover, they can enter the host bloodstream to influence systemic immune activity and affect respiratory tract microbial populations. Temperature and pH are typically stable throughout the gastrointestinal system, and the movement of bacteria is unidirectional and constantly altered by complicated physicochemical factors. Conversely, the lungs undergo frequent gaseous exchange with the external environment in order to maintain an abundance of oxygen and microorganisms [2]. Moreover, the absence of a physical barrier and the pressure and temperature gradient in the upper airways create bidirectional conditions for the movement and dynamic changes of microbes living in the lungs.

In several recent studies, researchers have described the dual role of lung bacteria in response to lung disease. Bingula et al. [119] demonstrated that bacteria and their fragments are taken up by DCs (dendritic cells) and macrophages through phagocytosis and subsequently migrate to the lung to control the immune response against the inflammatory response. Tsay et al. [120] showed that the gut microbiome can trigger an inflammatory response in the lung against bacterial pneumonia and promote neutrophil infiltration via TLR4 in mice. By producing genotoxins and DNA-reactive metabolites, generating free radicals, or regulating host immunocompetent cells, the microbiome can cause genome damage in host cells. Taken together, lung bacteria can behave as sophisticated and intervening ecosystems that control multiple pathogenic processes and maintain the physiological balance of the lung from two perspectives.

Despite the significant contribution of bacteria to lung homeostasis and immunoregulation, the orientation is not well defined; it is uncertain, depending on the context, whether lung pathology affects the microbial ecology, dysbiosis causes the development of the illness, or both. To appreciate the organization of the lung microbiome, the gut–lung axis, host–microbe interactions in the lung, and their function in the development of lung cancer, as well as how they may be exploited as diagnostic markers or prognostic indicators, further study is necessary. Liu et al. discovered considerably higher *Actinobacteria* at the phylum level as a potential lung cancer biomarker [121].

Moving forward, the presence of bacteria in the specific tissue can be considered as a biomarker. For instance, the combination of two bacterial biomarkers, namely, *Capnocytophaga* and *Veillonella*, showed excellent performance in the prognosis of squamous cell carcinoma (SCC) and adenocarcinoma (AC), which may also contribute to screening of lung cancer [23]. In addition, the influence of bacteria on anticancer drugs should be adequately considered. Given that the diversity of intestinal bacteria has a substantial influence on the bioactivity and therapeutic efficacy of the medicine, this opens the door for novel treatment techniques focused at restoring intestine eubiosis to increase the life of cancer patients.

## Figures and Tables

**Table 1:** Summary of lung microbiota and the interplays with pulmonary disease.

Taxa	Types	Sample Source or Sampling Method	Related Diseases	Potential Functions and Mechanism
*Prevotella* [29]	Gram-negative, anaerobes	BALF, human air brushes	Lung cancer	Upregulating lung cancer pathogenesis ERK and PI3K signaling pathways
*Veillonella* [36]	Gram-negative, anaerobes	BALF, lung tissues	Adenocarcinoma, Squamous cell carcinoma	Upregulating ERK and PI3K pathways; positively correlating with Th17 cells and neutrophils
*Pseudomonas* [35]	Gram-negative, aerobes	BALF, lung tissues	Lung adenocarcinoma	Colonizing in COPD patients epidemiologically; potentially indicating worse status
*Staphylococcus* [26]	Gram-positive, facultative anaerobes	BALF, lung tissues	Idiopathic pulmonary fibrosis	Inducing progression of pulmonary fibrosis through pneumolysin
*Sphingomonas* [37]	Gram-negative, aerobes	BALF, lung tissues	Adenocarcinoma	Increasing macrophage abundance and IFN-g level in the BAL
*Stenotrophomonas* [38]	Gram-negative, aerobes	BALF, lung tissues	Bronchiectasis	Linked with host genotype (fucosyltransferase 2 secretors)
*Actinobacillus* [39]	Gram-negative, facultative anaerobes	Human feces	Squamous cell carcinoma	Correlated with lung cancer status and stage
*Granulicatella* [35]	Gram-positive, facultative anaerobes	Human oral and sputum samples	Lung cancer, cystic fibrosis	Attributed to household coal burning exposures compared to healthy controls
*Acidovorax* [11]	Gram-negative, facultative anaerobes	Lung tissues	Lung cancer	Increasing mutation frequency of TP53
*Streptococcus* [40]	Gram-positive, facultative anaerobes	Lung cancer and normal tissues	Lung cancer	Inducing γδ T cells; promoting inflammation and lung cancer development

BALF: bronchoalveolar lavage fluid.

## Data Availability

Not applicable.

## References

[1] Gilbert J.A., Blaser M.J., Caporaso J.G., Jansson J.K., Lynch S.V., Knight R. (2018). Current understanding of the human microbiome. Nat. Med..

[2] Lozupone C.A., Stombaugh J.I., Gordon J.I., Jansson J.K., Knight R. (2012). Diversity, stability and resilience of the human gut microbiota. Nature.

[3] Gately S. (2019). Human Microbiota and Personalized Cancer Treatments: Role of Commensal Microbes in Treatment Outcomes for Cancer Patients. Cancer Treat. Res..

[4] Wang Y., Wu J., Cao Y. (2015). The extended spectrum β-lactamases (ESBL) and virulence genes of intestinal enteroaggregative Escherichia coli (EAEC) in healthy elderly individuals. Int. J. Clin. Exp. Med..

[5] Sepich-Poore G.D., Zitvogel L., Straussman R., Hasty J., Wargo J.A., Knight R. (2021). The microbiome and human cancer. Science.

[6] Zitvogel L., Daillère R., Roberti M.P., Routy B., Kroemer G. (2017). Anticancer effects of the microbiome and its products. Nat. Rev. Microbiol..

[7] Brandi G., Frega G. (2019). Microbiota: Overview and Implication in Immunotherapy-Based Cancer Treatments. Int. J. Mol. Sci..

[8] Iida N., Dzutsev A., Stewart C.A., Smith L., Bouladoux N., Weingarten R.A., Molina D.A., Salcedo R., Back T., Cramer S. (2013). Commensal bacteria control cancer response to therapy by modulating the tumor microenvironment. Science.

[9] Sivan A., Corrales L., Hubert N., Williams J.B., Aquino-Michaels K., Earley Z.M., Benyamin F.W., Lei Y.M., Jabri B., Alegre M.L. (2015). Commensal Bifidobacterium promotes antitumor immunity and facilitates anti-PD-L1 efficacy. Science.

[10] Dickson R.P., Huffnagle G.B. (2015). The Lung Microbiome: New Principles for Respiratory Bacteriology in Health and Disease. PLoS Pathog..

[11] Greathouse K.L., White J.R., Vargas A.J., Bliskovsky V.V., Beck J.A., von Muhlinen N., Polley E.C., Bowman E.D., Khan M.A., Robles A.I. (2018). Interaction between the microbiome and TP53 in human lung cancer. Genome Biol..

[12] Ramírez-Labrada A.G., Isla D., Artal A., Arias M., Rezusta A., Pardo J., Gálvez E.M. (2020). The Influence of Lung Microbiota on Lung Carcinogenesis, Immunity, and Immunotherapy. Trends Cancer.

[13] Gopalakrishnan V., Spencer C.N., Nezi L., Reuben A., Andrews M.C., Karpinets T.V., Prieto P.A., Vicente D., Hoffman K., Wei S.C. (2018). Gut microbiome modulates response to anti-PD-1 immunotherapy in melanoma patients. Science.

[14] Matson V., Fessler J., Bao R., Chongsuwat T., Zha Y., Alegre M.L., Luke J.J., Gajewski T.F. (2018). The commensal microbiome is associated with anti-PD-1 efficacy in metastatic melanoma patients. Science.

[15] Nejman D., Livyatan I., Fuks G., Gavert N., Zwang Y., Geller L.T., Rotter-Maskowitz A., Weiser R., Mallel G., Gigi E. (2020). The human tumor microbiome is composed of tumor type-specific intracellular bacteria. Science.

[16] Garrett W.S. (2019). The gut microbiota and colon cancer. Science.

[17] Shalapour S., Karin M. (2020). Cruel to Be Kind: Epithelial, Microbial, and Immune Cell Interactions in Gastrointestinal Cancers. Annu. Rev. Immunol..

[18] Tsay J.J., Wu B.G., Badri M.H., Clemente J.C., Shen N., Meyn P., Li Y., Yie T.A., Lhakhang T., Olsen E. (2018). Airway Microbiota Is Associated with Upregulation of the PI3K Pathway in Lung Cancer. Am. J. Respir. Crit. Care Med..

[19] Liu F., Li J., Guan Y., Lou Y., Chen H., Xu M., Deng D., Chen J., Ni B., Zhao L. (2019). Dysbiosis of the Gut Microbiome is associated with Tumor Biomarkers in Lung Cancer. Int. J. Biol. Sci..

[20] Zhao F., An R., Wang L., Shan J., Wang X. (2021). Specific Gut Microbiome and Serum Metabolome Changes in Lung Cancer Patients. Front. Cell. Infect. Microbiol..

[21] Huang D., Su X., Yuan M., Zhang S., He J., Deng Q., Qiu W., Dong H., Cai S. (2019). The characterization of lung microbiome in lung cancer patients with different clinicopathology. Am. J. Cancer Res..

[22] Lee S.H., Sung J.Y., Yong D., Chun J., Kim S.Y., Song J.H., Chung K.S., Kim E.Y., Jung J.Y., Kang Y.A. (2016). Characterization of microbiome in bronchoalveolar lavage fluid of patients with lung cancer comparing with benign mass like lesions. Lung Cancer.

[23] Yan X., Yang M., Liu J., Gao R., Hu J., Li J., Zhang L., Shi Y., Guo H., Cheng J. (2015). Discovery and validation of potential bacterial biomarkers for lung cancer. Am. J. Cancer Res..

[24] Scrima M., De Marco C., Fabiani F., Franco R., Pirozzi G., Rocco G., Ravo M., Weisz A., Zoppoli P., Ceccarelli M. (2012). Signaling networks associated with AKT activation in non-small cell lung cancer (NSCLC): New insights on the role of phosphatydil-inositol-3 kinase. PLoS ONE.

[25] Gustafson A.M., Soldi R., Anderlind C., Scholand M.B., Qian J., Zhang X., Cooper K., Walker D., McWilliams A., Liu G. (2010). Airway PI3K pathway activation is an early and reversible event in lung cancer development. Sci. Transl. Med..

[26] Segal L.N., Alekseyenko A.V., Clemente J.C., Kulkarni R., Wu B., Gao Z., Chen H., Berger K.I., Goldring R.M., Rom W.N. (2013). Enrichment of lung microbiome with supraglottic taxa is associated with increased pulmonary inflammation. Microbiome.

[27] Segal L.N., Clemente J.C., Tsay J.C., Koralov S.B., Keller B.C., Wu B.G., Li Y., Shen N., Ghedin E., Morris A. (2016). Enrichment of the lung microbiome with oral taxa is associated with lung inflammation of a Th17 phenotype. Nat. Microbiol..

[28] Dickson R.P., Erb-Downward J.R., Freeman C.M., McCloskey L., Falkowski N.R., Huffnagle G.B., Curtis J.L. (2017). Bacterial Topography of the Healthy Human Lower Respiratory Tract. mBio.

[29] Charlson E.S., Bittinger K., Haas A.R., Fitzgerald A.S., Frank I., Yadav A., Bushman F.D., Collman R.G. (2011). Topographical continuity of bacterial populations in the healthy human respiratory tract. Am. J. Respir. Crit. Care Med..

[30] Bassis C.M., Erb-Downward J.R., Dickson R.P., Freeman C.M., Schmidt T.M., Young V.B., Beck J.M., Curtis J.L., Huffnagle G.B. (2015). Analysis of the upper respiratory tract microbiotas as the source of the lung and gastric microbiotas in healthy individuals. mBio.

[31] Sampaio-Maia B., Caldas I.M., Pereira M.L., Pérez-Mongiovi D., Araujo R. (2016). The Oral Microbiome in Health and Its Implication in Oral and Systemic Diseases. Adv. Appl. Microbiol..

[32] Durack J., Huang Y.J., Nariya S., Christian L.S., Ansel K.M., Beigelman A., Castro M., Dyer A.M., Israel E., Kraft M. (2018). Bacterial biogeography of adult airways in atopic asthma. Microbiome.

[33] Carmody L.A., Zhao J., Schloss P.D., Petrosino J.F., Murray S., Young V.B., Li J.Z., LiPuma J.J. (2013). Changes in cystic fibrosis airway microbiota at pulmonary exacerbation. Ann. Am. Thorac. Soc..

[34] Bisgaard H., Hermansen M.N., Buchvald F., Loland L., Halkjaer L.B., Bønnelykke K., Brasholt M., Heltberg A., Vissing N.H., Thorsen S.V. (2007). Childhood asthma after bacterial colonization of the airway in neonates. N. Engl. J. Med..

[35] Yu G., Gail M.H., Consonni D., Carugno M., Humphrys M., Pesatori A.C., Caporaso N.E., Goedert J.J., Ravel J., Landi M.T. (2016). Characterizing human lung tissue microbiota and its relationship to epidemiological and clinical features. Genome Biol..

[36] Zheng Y., Fang Z., Xue Y., Zhang J., Zhu J., Gao R., Yao S., Ye Y., Wang S., Lin C. (2020). Specific gut microbiome signature predicts the early-stage lung cancer. Gut Microbes.

[37] Willner D., Haynes M.R., Furlan M., Schmieder R., Lim Y.W., Rainey P.B., Rohwer F., Conrad D. (2012). Spatial distribution of microbial communities in the cystic fibrosis lung. ISME J..

[38] Taylor S.L., Woodman R.J., Chen A.C., Burr L.D., Gordon D.L., McGuckin M.A., Wesselingh S., Rogers G.B. (2017). FUT2 genotype influences lung function, exacerbation frequency and airway microbiota in non-CF bronchiectasis. Thorax.

[39] Gomes S., Cavadas B., Ferreira J.C., Marques P.I., Monteiro C., Sucena M., Sousa C., Vaz Rodrigues L., Teixeira G., Pinto P. (2019). Profiling of lung microbiota discloses differences in adenocarcinoma and squamous cell carcinoma. Sci. Rep..

[40] Grasso F., Frisan T. (2015). Bacterial Genotoxins: Merging the DNA Damage Response into Infection Biology. Biomolecules.

[41] Alexandrov L.B., Ju Y.S., Haase K., Van Loo P., Martincorena I., Nik-Zainal S., Totoki Y., Fujimoto A., Nakagawa H., Shibata T. (2016). Mutational signatures associated with tobacco smoking in human cancer. Science.

[42] Mao Q., Jiang F., Yin R., Wang J., Xia W., Dong G., Ma W., Yang Y., Xu L., Hu J. (2018). Interplay between the lung microbiome and lung cancer. Cancer Lett..

[43] Chen J., Domingue J.C., Sears C.L. (2017). Microbiota dysbiosis in select human cancers: Evidence of association and causality. Semin. Immunol..

[44] Garrett W.S. (2015). Cancer and the microbiota. Science.

[45] Zhang W.Q., Zhao S.K., Luo J.W., Dong X.P., Hao Y.T., Li H., Shan L., Zhou Y., Shi H.B., Zhang Z.Y. (2018). Alterations of fecal bacterial communities in patients with lung cancer. Am. J. Transl. Res..

[46] Gui Q., Li H., Wang A., Zhao X., Tan Z., Chen L., Xu K., Xiao C. (2020). The association between gut butyrate-producing bacteria and non-small-cell lung cancer. J. Clin. Lab. Anal..

[47] Zhang J., Guo Z., Xue Z., Sun Z., Zhang M., Wang L., Wang G., Wang F., Xu J., Cao H. (2015). A phylo-functional core of gut microbiota in healthy young Chinese cohorts across lifestyles, geography and ethnicities. ISME J..

[48] Budden K.F., Gellatly S.L., Wood D.L., Cooper M.A., Morrison M., Hugenholtz P., Hansbro P.M. (2017). Emerging pathogenic links between microbiota and the gut-lung axis. Nat. Rev. Microbiol..

[49] Wu S., Rhee K.J., Albesiano E., Rabizadeh S., Wu X., Yen H.R., Huso D.L., Brancati F.L., Wick E., McAllister F. (2009). A human colonic commensal promotes colon tumorigenesis via activation of T helper type 17 T cell responses. Nat. Med..

[50] Li R., Zhou R., Wang H., Li W., Pan M., Yao X., Zhan W., Yang S., Xu L., Ding Y. (2019). Gut microbiota-stimulated cathepsin K secretion mediates TLR4-dependent M2 macrophage polarization and promotes tumor metastasis in colorectal cancer. Cell Death Differ..

[51] Jungnickel C., Schmidt L.H., Bittigkoffer L., Wolf L., Wolf A., Ritzmann F., Kamyschnikow A., Herr C., Menger M.D., Spieker T. (2017). IL-17C mediates the recruitment of tumor-associated neutrophils and lung tumor growth. Oncogene.

[52] Gollwitzer E.S., Saglani S., Trompette A., Yadava K., Sherburn R., McCoy K.D., Nicod L.P., Lloyd C.M., Marsland B.J. (2014). Lung microbiota promotes tolerance to allergens in neonates via PD-L1. Nat. Med..

[53] Ichinohe T., Pang I.K., Kumamoto Y., Peaper D.R., Ho J.H., Murray T.S., Iwasaki A. (2011). Microbiota regulates immune defense against respiratory tract influenza A virus infection. Proc. Natl. Acad. Sci. USA.

[54] Rosshart S.P., Vassallo B.G., Angeletti D., Hutchinson D.S., Morgan A.P., Takeda K., Hickman H.D., McCulloch J.A., Badger J.H., Ajami N.J. (2017). Wild Mouse Gut Microbiota Promotes Host Fitness and Improves Disease Resistance. Cell.

[55] Johansson M.E., Jakobsson H.E., Holmén-Larsson J., Schütte A., Ermund A., Rodríguez-Piñeiro A.M., Arike L., Wising C., Svensson F., Bäckhed F. (2015). Normalization of Host Intestinal Mucus Layers Requires Long-Term Microbial Colonization. Cell Host Microbe.

[56] Mantis N.J., Rol N., Corthésy B. (2011). Secretory IgA’s complex roles in immunity and mucosal homeostasis in the gut. Mucosal Immunol..

[57] Dzutsev A., Goldszmid R.S., Viaud S., Zitvogel L., Trinchieri G. (2015). The role of the microbiota in inflammation, carcinogenesis, and cancer therapy. Eur. J. Immunol..

[58] Huda M.N., Lewis Z., Kalanetra K.M., Rashid M., Ahmad S.M., Raqib R., Qadri F., Underwood M.A., Mills D.A., Stephensen C.B. (2014). Stool microbiota and vaccine responses of infants. Pediatrics.

[59] Elangovan S., Pathania R., Ramachandran S., Ananth S., Padia R.N., Lan L., Singh N., Martin P.M., Hawthorn L., Prasad P.D. (2014). The niacin/butyrate receptor GPR109A suppresses mammary tumorigenesis by inhibiting cell survival. Cancer Res..

[60] Ngo V.N., Young R.M., Schmitz R., Jhavar S., Xiao W., Lim K.H., Kohlhammer H., Xu W., Yang Y., Zhao H. (2011). Oncogenically active MYD88 mutations in human lymphoma. Nature.

[61] Chen G.Y., Shaw M.H., Redondo G., Núñez G. (2008). The innate immune receptor Nod1 protects the intestine from inflammation-induced tumorigenesis. Cancer Res..

[62] Ali T., Kaitha S., Mahmood S., Ftesi A., Stone J., Bronze M.S. (2013). Clinical use of anti-TNF therapy and increased risk of infections. Drug Healthc. Patient Saf..

[63] Chen L., Wilson J.E., Koenigsknecht M.J., Chou W.C., Montgomery S.A., Truax A.D., Brickey W.J., Packey C.D., Maharshak N., Matsushima G.K. (2017). Erratum: NLRP12 attenuates colon inflammation by maintaining colonic microbial diversity and promoting protective commensal bacterial growth. Nat. Immunol..

[64] Huang Y.J., Nariya S., Harris J.M., Lynch S.V., Choy D.F., Arron J.R., Boushey H. (2015). The airway microbiome in patients with severe asthma: Associations with disease features and severity. J. Allergy Clin. Immunol..

[65] Winter S.E., Winter M.G., Xavier M.N., Thiennimitr P., Poon V., Keestra A.M., Laughlin R.C., Gomez G., Wu J., Lawhon S.D. (2013). Host-derived nitrate boosts growth of E. coli in the inflamed gut. Science.

[66] Winter S.E., Baumler A.J. (2014). Dysbiosis in the inflamed intestine: Chance favors the prepared microbe. Gut Microbes.

[67] Huffnagle G.B., Dickson R.P., Lukacs N.W. (2017). The respiratory tract microbiome and lung inflammation: A two-way street. Mucosal Immunol..

[68] Du X., Wei J., Tian D., Wu M., Yan C., Hu P., Wu X., Yang W., Yin T. (2020). miR-182-5p contributes to intestinal injury in a murine model of Staphylococcus aureus pneumonia-induced sepsis via targeting surfactant protein D. J. Cell Physiol..

[69] Zhang H., Yeh C., Jin Z., Ding L., Liu B.Y., Zhang L., Dannelly H.K. (2018). Prospective study of probiotic supplementation results in immune stimulation and improvement of upper respiratory infection rate. Synth. Syst. Biotechnol..

[70] Van Best N., Rolle-Kampczyk U., Schaap F.G., Basic M., Olde Damink S.W.M., Bleich A., Savelkoul P.H.M., von Bergen M., Penders J., Hornef M.W. (2020). Bile acids drive the newborn’s gut microbiota maturation. Nat. Commun..

[71] Vanegas S.M., Meydani M., Barnett J.B., Goldin B., Kane A., Rasmussen H., Brown C., Vangay P., Knights D., Jonnalagadda S. (2017). Substituting whole grains for refined grains in a 6-wk randomized trial has a modest effect on gut microbiota and immune and inflammatory markers of healthy adults. Am. J. Clin. Nutr..

[72] Anand S., Mande S.S. (2018). Diet, Microbiota and Gut-Lung Connection. Front. Microbiol..

[73] Wypych T.P., Wickramasinghe L.C., Marsland B.J. (2019). The influence of the microbiome on respiratory health. Nat. Immunol..

[74] Keren N., Konikoff F.M., Paitan Y., Gabay G., Reshef L., Naftali T., Gophna U. (2015). Interactions between the intestinal microbiota and bile acids in gallstones patients. Environ. Microbiol. Rep..

[75] Qian G., Jiang W., Zou B., Feng J., Cheng X., Gu J., Chu T., Niu C., He R., Chu Y. (2018). LPS inactivation by a host lipase allows lung epithelial cell sensitization for allergic asthma. J. Exp. Med..

[76] Cribbs S.K., Uppal K., Li S., Jones D.P., Huang L., Tipton L., Fitch A., Greenblatt R.M., Kingsley L., Guidot D.M. (2016). Correlation of the lung microbiota with metabolic profiles in bronchoalveolar lavage fluid in HIV infection. Microbiome.

[77] Gao B., Gallagher T., Zhang Y., Elbadawi-Sidhu M., Lai Z., Fiehn O., Whiteson K.L. (2018). Tracking Polymicrobial Metabolism in Cystic Fibrosis Airways: Pseudomonas aeruginosa Metabolism and Physiology Are Influenced by Rothia mucilaginosa-Derived Metabolites. mSphere.

[78] Trompette A., Gollwitzer E.S., Pattaroni C., Lopez-Mejia I.C., Riva E., Pernot J., Ubags N., Fajas L., Nicod L.P., Marsland B.J. (2018). Dietary Fiber Confers Protection against Flu by Shaping Ly6c(-) Patrolling Monocyte Hematopoiesis and CD8(+) T Cell Metabolism. Immunity.

[79] Eid N., Osmanova H., Natchez C., Walton G., Costabile A., Gibson G., Rowland I., Spencer J.P. (2015). Impact of palm date consumption on microbiota growth and large intestinal health: A randomised, controlled, cross-over, human intervention study. Br. J. Nutr..

[80] Hirayama K., Baranczewski P., Akerlund J.E., Midtvedt T., Möller L., Rafter J. (2000). Effects of human intestinal flora on mutagenicity of and DNA adduct formation from food and environmental mutagens. Carcinogenesis.

[81] Vanhaecke L., Knize M.G., Noppe H., De Brabander H., Verstraete W., Van de Wiele T. (2008). Intestinal bacteria metabolize the dietary carcinogen 2-amino-1-methyl-6-phenylimidazo[4,5-b]pyridine following consumption of a single cooked chicken meal in humans. Food Chem. Toxicol. Int. J. Publ. Br. Ind. Biol. Res. Assoc..

[82] Tomasello G., Tralongo P., Damiani P., Sinagra E., Di Trapani B., Zeenny M.N., Hussein I.H., Jurjus A., Leone A. (2014). Dismicrobism in inflammatory bowel disease and colorectal cancer: Changes in response of colocytes. World J. Gastroenterol..

[83] Maier I., Berry D.M., Schiestl R.H. (2014). Intestinal microbiota reduces genotoxic endpoints induced by high-energy protons. Radiat. Res..

[84] Van Nood E., Vrieze A., Nieuwdorp M., Fuentes S., Zoetendal E.G., de Vos W.M., Visser C.E., Kuijper E.J., Bartelsman J.F., Tijssen J.G. (2013). Duodenal infusion of donor feces for recurrent Clostridium difficile. N. Engl. J. Med..

[85] Belizário J.E., Napolitano M. (2015). Human microbiomes and their roles in dysbiosis, common diseases, and novel therapeutic approaches. Front. Microbiol..

[86] Petersen C., Round J.L. (2014). Defining dysbiosis and its influence on host immunity and disease. Cell. Microbiol..

[87] Tulic M.K., Piche T., Verhasselt V. (2016). Lung-gut cross-talk: Evidence, mechanisms and implications for the mucosal inflammatory diseases. Clin. Exp. Allergy J. Br. Soc. Allergy Clin. Immunol..

[88] Laroumagne S., Salinas-Pineda A., Hermant C., Murris M., Gourraud P.A., Do C., Segonds C., Didier A., Mazières J. (2011). Incidence and characteristics of bronchial colonisation in patient with lung cancer: A retrospective study of 388 cases. Rev. Mal. Respir..

[89] Sobhani I., Bergsten E., Couffin S., Amiot A., Nebbad B., Barau C., de’Angelis N., Rabot S., Canoui-Poitrine F., Mestivier D. (2019). Colorectal cancer-associated microbiota contributes to oncogenic epigenetic signatures. Proc. Nat. Acad. Sci. USA.

[90] Rosignoli P., Fabiani R., De Bartolomeo A., Spinozzi F., Agea E., Pelli M.A., Morozzi G. (2001). Protective activity of butyrate on hydrogen peroxide-induced DNA damage in isolated human colonocytes and HT29 tumour cells. Carcinogenesis.

[91] Pushalkar S., Hundeyin M., Daley D., Zambirinis C.P., Kurz E., Mishra A., Mohan N., Aykut B., Usyk M., Torres L.E. (2018). The Pancreatic Cancer Microbiome Promotes Oncogenesis by Induction of Innate and Adaptive Immune Suppression. Cancer Discov..

[92] Vitiello G.A., Cohen D.J., Miller G. (2019). Harnessing the Microbiome for Pancreatic Cancer Immunotherapy. Trends Cancer.

[93] Sender R., Fuchs S., Milo R. (2016). Revised Estimates for the Number of Human and Bacteria Cells in the Body. PLoS Biol..

[94] Hibbing M.E., Fuqua C., Parsek M.R., Peterson S.B. (2010). Bacterial competition: Surviving and thriving in the microbial jungle. Nat. Rev. Microbiol..

[95] Guerra L., Cortes-Bratti X., Guidi R., Frisan T. (2011). The biology of the cytolethal distending toxins. Toxins.

[96] Nougayrède J.P., Homburg S., Taieb F., Boury M., Brzuszkiewicz E., Gottschalk G., Buchrieser C., Hacker J., Dobrindt U., Oswald E. (2006). Escherichia coli induces DNA double-strand breaks in eukaryotic cells. Science.

[97] Frisan T. (2016). Bacterial genotoxins: The long journey to the nucleus of mammalian cells. Biochim. Biophys. Acta.

[98] Kwa M., Plottel C.S., Blaser M.J., Adams S. (2016). The Intestinal Microbiome and Estrogen Receptor-Positive Female Breast Cancer. J. Natl. Cancer Inst..

[99] Yaghoobi H., Bandehpour M., Kazemi B. (2016). Apoptotic Effects of the B Subunit of Bacterial Cytolethal Distending Toxin on the A549 Lung Cancer Cell Line. Asian Pac. J. Cancer Prev. APJCP.

[100] Travaglione S., Fabbri A., Fiorentini C. (2008). The Rho-activating CNF1 toxin from pathogenic E. coli: A risk factor for human cancer development?. Infect. Agents Cancer.

[101] Nesić D., Hsu Y., Stebbins C.E. (2004). Assembly and function of a bacterial genotoxin. Nature.

[102] Carbonero F., Benefiel A.C., Alizadeh-Ghamsari A.H., Gaskins H.R. (2012). Microbial pathways in colonic sulfur metabolism and links with health and disease. Front. Physiol..

[103] Rubinstein M.R., Wang X., Liu W., Hao Y., Cai G., Han Y.W. (2013). Fusobacterium nucleatum promotes colorectal carcinogenesis by modulating E-cadherin/β-catenin signaling via its FadA adhesin. Cell Host Microbe.

[104] Burns M.B., Lynch J., Starr T.K., Knights D., Blekhman R. (2015). Virulence genes are a signature of the microbiome in the colorectal tumor microenvironment. Genome Med..

[105] Chen Z.Y., Xiao H.W., Dong J.L., Li Y., Wang B., Fan S.J., Cui M. (2021). Gut Microbiota-Derived PGF2α Fights against Radiation-Induced Lung Toxicity through the MAPK/NF-κB Pathway. Antioxidants.

[106] Kanwal R., Gupta K., Gupta S. (2015). Cancer epigenetics: An introduction. Methods Mol. Biol..

[107] Licchesi J.D., Westra W.H., Hooker C.M., Herman J.G. (2008). Promoter hypermethylation of hallmark cancer genes in atypical adenomatous hyperplasia of the lung. Clin. Cancer Res. Off. J. Am. Assoc. Cancer Res..

[108] Hamon M.A., Cossart P. (2008). Histone modifications and chromatin remodeling during bacterial infections. Cell Host Microbe.

[109] Bierne H., Hamon M., Cossart P. (2012). Epigenetics and bacterial infections. Cold Spring Harb. Perspect. Med..

[110] Arbibe L. (2008). Immune subversion by chromatin manipulation: A ‘new face’ of host-bacterial pathogen interaction. Cell. Microbiol..

[111] Chang P.V., Hao L., Offermanns S., Medzhitov R. (2014). The microbial metabolite butyrate regulates intestinal macrophage function via histone deacetylase inhibition. Proc. Nat. Acad. Sci. USA.

[112] Zur Bruegge J., Einspanier R., Sharbati S. (2017). A Long Journey Ahead: Long Non-coding RNAs in Bacterial Infections. Front. Cell. Infect. Microbiol..

[113] Hu Y., Ren S., He Y., Wang L., Chen C., Tang J., Liu W., Yu F. (2020). Possible Oncogenic Viruses Associated with Lung Cancer. OncoTargets Ther..

[114] Lieberman P.M. (2008). Chromatin organization and virus gene expression. J. Cell. Physiol..

[115] Krautkramer K.A., Rey F.E., Denu J.M. (2017). Chemical signaling between gut microbiota and host chromatin: What is your gut really saying?. J. Biol. Chem..

[116] Rossi M., Amaretti A., Raimondi S. (2011). Folate production by probiotic bacteria. Nutrients.

[117] Zempleni J., Teixeira D.C., Kuroishi T., Cordonier E.L., Baier S. (2012). Biotin requirements for DNA damage prevention. Mutat. Res..

[118] Wong-Rolle A., Wei H.K., Zhao C., Jin C. (2021). Unexpected guests in the tumor microenvironment: Microbiome in cancer. Protein Cell.

[119] Bingula R., Filaire M., Radosevic-Robin N., Bey M., Berthon J.Y., Bernalier-Donadille A., Vasson M.P., Filaire E. (2017). Desired Turbulence? Gut-Lung Axis, Immunity, and Lung Cancer. J. Oncol..

[120] Tsay T.B., Yang M.C., Chen P.H., Hsu C.M., Chen L.W. (2011). Gut flora enhance bacterial clearance in lung through toll-like receptors 4. J. Biomed. Sci..

[121] Liu S., Li E., Sun Z., Fu D., Duan G., Jiang M., Yu Y., Mei L., Yang P., Tang Y. (2019). Altered gut microbiota and short chain fatty acids in Chinese children with autism spectrum disorder. Sci. Rep..

